# Neonatal tetanus in South Sudan: a case series

**DOI:** 10.4314/ahs.v24i3.11

**Published:** 2024-09

**Authors:** Justin B Tongun, Amanda BB Madison, Joseph D Lako, Kenneth L Sube

**Affiliations:** 1 Al Sabah Children Hospital, Juba, South Sudan; 2 Department of Pediatric and Child Health, School of Medicine, University of Juba, Juba, South Sudan; 3 Health and Social Sciences Research Institute, Juba, South Sudan (HSSRI-SS); 4 School of Applied and Industrial Sciences, University of Juba, Juba, South Sudan; 5 Department of Ophthalmology, School of Medicine, University of Juba, Juba, South Sudan; 6 South University of Medicine, Science and Technology (SUMST), Juba, South Sudan

**Keywords:** Neonatal tetanus, low-income countries, neonates, Al sabah children hospital, South Sudan

## Abstract

**Introduction:**

Neonatal tetanus is a life-threatening disease of public health importance; it is yet to be eliminated and is still occurring in South Sudan. It is caused by a neurotoxin from a bacterium Clostridium tetani whose spores exist in the environment. Compared to high-income countries, most low-income countries lack intensive care units, and magnesium sulfate shown to improve neonatal tetanus outcomes.

**Objectives:**

We aimed to determine the outcome of neonatal tetanus in Al Sabah Children's Hospital, Juba, South Sudan.

**Methods:**

We conducted a case series study in Al Sabah Children's Hospital in Juba, South Sudan. It described three neonates who presented within the first seven days of life with a history of excessive crying, inability to breastfeed, and tetanic muscle spasms when stimulated. They had signs of respiratory distress, fever, and labile heart rates. We made a diagnosis of neonatal tetanus with autonomic dysfunction, and started them on phenobarbitone because diazepam was not available, oxygen, and antibiotics. However, tetanus immune globulin and anti-tetanus serum were not available.

**Results:**

Unfortunately, all three neonates died within 24 hours of admission.

**Conclusion:**

Our primary focus as a nation is to improve access to quality health services and the prevention of neonatal tetanus through encouraging appropriate antenatal care, facility delivery, clean delivery and healthy umblical cord care practices, and tetanus vaccinations in teenage girls and young adults. When prevention fails, there is a need for neonatal intensive care with recommended medicines to significantly decrease mortality in this preventable tragic illness.

## Introduction

Globally, the burden of tetanus has gradually declined from an estimated 800,000 in the 1980s to 50, 000 in 2015^1^,^2^. However, neonatal tetanus remains a common cause of high morbidity and mortality in low-income - countries^3^,^4^. Tetanus is a fatal disease caused by a neurotoxin produced by the bacterium Clostridium tetani. The bacterium spores are often found in the environment globally. These spores gain access through a breached skin such as contaminated wounds or tissue injuries, which eventually develop into organisms in an anaerobic environment releasing tetanus toxin^5^, ^6^ into the susceptible host system. Tetanus can present in different forms including, local tetanus; cephalic tetanus; generalized tetanus in adults, and neonatal tetanus^7^. This condition may arise due to different contributing factors, especially in low-resource settings.

Factors associated with transmission of the bacterium include delivery in an unclean environment; cutting of umbilical cord with a non-sterile instrument, application of cow dung or other substances on umbilical cord, and other unsafe traditional practices^8^. A case definition of neonatal tetanus encompassed thfollowing: any neonate with a normal ability to suck and cry during the first two days of life; and who between 3 and 28 days of life cannot suck normally and has generalized muscle rigidity and/or tetanic muscle spasms, which is associated with neonatal mortality^8^. Neonatal tetanus mortality rate can be as high as 100%. The mortality is high when symptoms start in less than seven days of life aggravated by autonomic nervous dysfunction i.e. unstable hypertension and heart rate, and spasm of respiratory muscle leading to respiratory failure. In a better setting, supportive care and treatment in neonatal intensive care unit (NICU) can decrease mortality.

Treatment of neonatal tetanus needs the administration of 500 IU of tetanus immunoglobulin, Metronidazole for 14 days or Crystalline Penicillin for 10-14 days as second line of treatment when the first line is unavailiable. In addition, the newborn can benefit from supportive care and intensive care unit (ICU) intervention. However, since it is not possible to eradicate it from the environment, prevention through vaccination with tetanus containing vaccine (TTCV), clean delivery practices, and healthy umbilical cord care remain the best options for preventing infection and eliminating mortality due to Tetanus^8^.

According to a Cochrane database systematic review, several modalities have been associated with prevention of neonatal tetanus like aseptic delivery, cord care, and vaccination of pregnant or non-pregnant women 9. The World Health Organization (WHO) recommends six doses of TTCV as follows: three infant doses, three boosters' doses at 12-23 months, 4-7 years, and 9-15 years. In addition, pregnant women and their babies remain protected if the mother received six doses of TTCV 8.

This study aimed to determine outcome of newborns diagnosed with neonatal tetanus in Al Sabah Children Hospital in Juba, South Sudan.

## Methods

**Study site:** This study was conducted in Al Sabah Children's Hospital, a tertiary referral hospital in Juba, South Sudan. The study described three cases of newborns diagnosed with neonatal tetanus.

**Case one:** A four-day old female, delivered at full term through a normal spontaneous vaginal delivery (SVD) at home. She cried immediately, had no complications at birth, and breastfed soon after birth. On day four of life, the mother noticed excessive crying. On the fifth day, she noticed tightness of lips, refusal to breastfeed, episodes of muscle spasms, and stiffness of the body when touched. These symptoms worsened over 6 hours on fift day of life, which prompted the mother to seek care in the hospital. In addition to the baby's history, the mother disclosed that she did not attend antenatal care (ANC) and did not receive TTCV or similar vaccine in childhood and during teenage years. Following delivery, the mother reported applying burned matchstick power mixed with oil on the umbilical stump.

**Examination:** the baby looked ill; temperature 36.4°C, no jaundice; no pallor on palms and mucus membrane; no finger clubbing; no central cyanosis; no lower limbs edema, no lymphadenopathy, and no dysmorphic features. She was lethargic with locked jaw, had abdominal muscle rigidity; generalized tetanic muscle spasms when touched. In addition, she had wet umbilicus stump covered with black particles. Further, HR 171 beats per minute, RR 63 breaths per minute with bronco vesicular breath sound and transmitted sounds, and SPO2 60-83% at room air. We diagnosed neonatal tetanus with differential diagnosis of early neonatal sepsis, and meningitis.

**Investigations findings:** Normal complete blood count (CBC), normal renal function, normal electrolytes, and negative blood smear for malaria. Given her unstable state, we did not do lumbar puncture for cerebral spinal fluid (CSF) analysis. No blood culture done since the service was not available in the hospital.

### Treatment

1. **Supportive care:** this included oxygen therapy 0.5 L/min, nasogastric (NG) tube inserted for feeding and 120 ml per kilogram/day of expressed breast milk (EBM) every 2 hours.
2. **Wound care:** cleaned the umbilical cord with hydrogen peroxide and applied chlorhexidine.
3. **Spasms control:** a loading dose of IV Phenobarbital 20mg/kg and maintenance of 2.5mg/kg 12 hourly. However, Diazepam and Magnesium sulfate were not available. We continued monitoring vital signs, frequency, and intensity of spasms.
4. **Tetanus immunoglobulin:** not given because it was not available.

Unfortunately, she died three hours after admission.

Case two: A seven-day-old male, full termed, delivered by normal SVD to a para two mother in a lower health facility. The baby cried immediately and no complications reported following delivery. With the current pregnancy, the mother reported receiving one dose of TTCV during ANC visit. She also remembered receiving two doses of TTCV with her first baby. However, she did not recall receiving TTCV in childhood and during teenage years. The mother reported that grandmother applied burned matchstick powder mixed with oil and other substances on the cord soon after birth. On day six of life at 7:22 PM, the mother brought the baby to hospital with complains of high-grade fever; refusal to breastfeed, and generalized tetanic muscle spasms.

### Examination

General examination revealed a sick looking neonate with axillary temperature of 37.9°C, there was no jaundice, no pallor of mucus membrane, and no dysmorphic features, but some dehydration.

**Systemic examinations showed the followings:**

Central nervous system: the baby was lethargic, with normal anterior fontanel, and increased tone.
Muscular skeletal system: he had tight lips, and muscle spasm when touched.

**Respiratory system:** the baby was in respiratory distress, fast breathing with RR=70 cycles/minute, bilateral equal air entry and coarse crepitation, and SPO2 92%,

Cardiovascular system: HR of 102 b/m, heart S1 and S2 were normal, and no murmur.

**Abdominal examination:** the abdomen was of normal fullness, moved with respiration, and the umbilicus had purulent dark discharge. Muscle spasm made it difficult to palpate for organs and ausculation revealed normal bowel sounds.

**Investigations:** complete blood count showed high white blood cell, negative blood smear for malaria, and normal electrolytes as well as renal function test.

**Diagnosis:** neonatal tetanus with differentials of neonatal sepsis, encephalitis, and meningitis

### Treatment

1. **Supportive care:** NG tube for feeding and 120ml[J2]/kg/24 hours of EBM given two hourly (Neonatal requirement for day 7 of life). Oxygen therapy 0.5 L/min, 10% dextrose 2 ml/kg bolus.
2. **Wound care:** we cleaned the umbilical cord with hydrogen peroxide and dressed it.
3. **Antibiotics:** IV Gentamycin 7.5mg/kg 12 hourly, IV Ampicillin 50mg/kg 12 hourly, IV Metronidazole.
4. **Spasm control:** loading dose of IV[J3] Phenobarbital at 20 mg/kg and maintenance of 2.5mg/kg/day
5. **Tetanus toxoid immunoglobulin:** not given because it was not available.

**Note:** diazepam was not available at the time of admission.

Despite the above treatment, the neonate died within 24 hours from the initiation of care.

### Case 3

A Seven-day old male delivered at full term by SVD to a peasant mother in a primary health care center. The baby cried immediately after birth with no complications and breastfed within the first hour obirth. Mother reported that she cleaned the cord with warm salty water and applied burned matchstick powder mixed with sesame oil to facilitate early cord detachment. On day seven of life, the neonate presented to the hospital with high-grade fever, refusal to breastfeed, excessive crying, locked jaw and episodic provoked spasms. The mother is Para 3+0 who attended ANC two times but did not receive TTCV because the vaccine was not available. She remembered receiving three doses of TTCV in the first pregnancy and two doses in the second pregnancy. She did not receive similar vaccine during teenage years. In addition, the mother reported that she tested HIV negative but had had episodes of urinary tract infections and malaria during pregnancy

On examination, the baby was ill looking, irritable, with provoked generalized tetanic muscle spasms when touched. He had fast breathing RR of 72 b/m, SPO2 63-79% at room air, temperature of 38.10C. He had bilateral equal air entry and normal breaths sounds. Normal heart rate 132 b/m, normal S1 and S2, and no murmur.

### Laboratory investigations

CBC showed normal findings and blood smear was negative for malaria parasites. The baby had normal renal function indices and electrolytes. We did not do lumbar puncture for CSF analysis because he was not in stable state. Lastly, no blood culture done because this service was not available. We made a diagnosis of neonatal tetanus and differential diagnosis of meningitis and neonatal sepsis.

### Management

1. **Supportive care:** oxygen therapy 0.5 L/min, NG) tube for feeding was inserted and 2 hourly EBM of 150ml/kg/24 hours was given
2. **IV antibiotics[J4]:** Ampicillin 50mg/kg 12 hourly, Gentamycin 5mg/kg 12 hourly and metronidazole 7.5mg/kg.
3. **Spasms control:** loading dose of IV Phenobarbital 20 mg/kg and maintenance of 2.5mg/kg 12 hourly.
4. **Cord care was done:** Diazepam, magnesium sulfate, and tetanus immunoglobulin were not availability during admission.

Unfortunately, the neonate died after six hours of treatment.

## Discussion

The global neonatal mortality rate has decreased from 37 deaths per 1,000 to 19 in 2016. Yet, it remains high in Great Lakes region and other low-income countries 10.

In this series, we reported on three neonates diagnosed with neonatal tetanus. However, all died within 24 hours of admission. This could have been due to the absence of intensive care and medical modalities including diazepam, magnesium sulfate, tetanus immune globulin, and anti-tetanus serum in our hospital. According to the WHO position paper, these treatment modalities have been demonstrated to decrease neonatal tetanus mortality 11. In addition, a study by Khan R, et al.^12^ reported an increase in case fatality of neonatal tetanus of up 100 % when medical treatment is absent. Further, evidence showed that neonatal tetanus mortality decreases from 60 % to 10% when hospital care as well as intensive care facilities are available 12.

Our result is comparable to that of other studies in low-resource settings, which reported high mortality of neonatal tetanus due to a lack of modern treatment modalities such as neuromuscular blockade and invasive ventilation 3, 14. On the other hand, evidence in high-resource settings showed that when benzodiazepines fail to control spasms, ventilation, and neuromuscular blockade are instituted which results in a better outcome^15^. These management interventions control respiratory muscle spasms known to reduce the risk of death due to respiratory muscle failure, which is a major cause of death in neonatal tetanus. However, these management modalities are not available in our setting. In this series, all three neonates presented with symptoms in the first week of life. Early presentation has been associated with high neonatal tetanus mortality as reported in a study in Vietnamese infants^15^. The newborns in our study presented with hyperpyrexia, tachypnea, unstable heart rate, respiratory distress, and tetanic muscle spasms. These clinical features have been associated with high mortality in infants with neonatal tetanus as reported in a study on incidence and risk factors for neonatal tetanus in Kilifi County^16^.

The neonates in our study had wet and soiled umbilical stumps, which could be the likely route of entry of *C. tetani*. In all the cases, mothers reported the use of burned matchstick power mixed with sesame oil presumed to enhance the detachment of the stumps from the umbilicus. This mixture could be the likely source of contamination of the cord and infection. A study by Raza S et, al. found that traditional home practices still exist during home deliveries 17. The same study reported application of harmful substances to umbilical stumps in areas within big urban cities with access to better healthcare services. This practice increases the fatality rate to 100% regardless of whether the deliveries take place at home or health care facilities^17^.

Prevention of neonatal tetanus is the best modality using two or more doses of tetanus toxoid vaccine given to the mother^8^. This is associated with a reduction of neonatal tetanus deaths by 94% 8. This benefit is a result of trans-placental transfer of antibodies from the mother to the fetus during pregnancy as reported in WHO guidelines 18. Other initiatives designed for the elimination of maternal and neonatal tetanus use strategies of immunization, clean delivery, and healthy cord care practices^19^. These strategies have contributed to the reduction of maternal and neonatal tetanus prevalence globally. According to the WHO^8^, the ideal administration of TTCV to mothers include several doses. First dose is at first contact with a health facility, which results in no protection, the second dose is one month after the first dose, which gives one-year protection. Five years protection is achieved when a mother received a third dose six months from the second dose, the fourth dose is given one year from the third dose and gives 10 years of protection, and the fifth dose is given one year from the fourth dose, which translates to protection for life. This strategy could eliminate the prevalence of neonatal tetanus when coverage rises to over 80%.

In this study, the mother in case one did not receive tetanus toxoid-containing vaccine, and thus could not confer protection to her neonate. On the other hand, the mother in case two received three doses of tetanus vaccine while that in case three received five doses. Ideally, these are adequate doses to confer protection against tetanus, sadly, their neonates still came down with tetanus. The possible explanation could be that the tetanus vaccine given to these mothers might have been poorly stored or expired. This could also be due to malaria infection in these mothers that likely affected the transfer of tetanus antibodies to the fetus during pregnancy. This is in agreement with a study by Cumberland P et, al. that found maternal HIV infection and placental malaria reduce trans placental antibody transfer to the fetus and tetanus antibody levels in newborns in Kenya 19.

It is worth mentioning that Sub-Saharan Africa has the highest prevalence of neonatal mortality 38% among all the regions 10. Countries in the Great Lakes namely Burundi, Democratic Republic of Congo, Kenya, Rwanda, Tanzania, and Uganda contribute to this high prevalence. Possible explanation include political instability, human resources, poor quality of health care services, insufficient health financing, poor socioeconomic status, low standard of health delivery service, and conflict^10^. Among them, the Democratic Republic of Congo had the highest neonatal mortality rate of 19 per 1000 births.

We could not find studies on neonatal tetanus in South Sudan. This case series might be the first to expose part of the problem. However, it is not representative of the whole country since it is a report of one hospital.

## Conclusion

Our primary focus as a nation should be to improve access to quality health services and the prevention of neonatal tetanus through encouraging appropriate ANC, facility delivery, clean delivery and healthy cord care practices, and tetanus vaccinations in our teenage girls and young adults. However, when prevention fails and a neonate presents with tetanus, there is a need to have intensive care with recommended medicines and support that should significantly decrease mortality in this preventable tragic illness.

## Data Availability

All data supporting our findings are available from the authors upon request.

## Declarations

Ethics approval and consent to participate was not needed for this case report.
